# Microaggressions towards lesbian and transgender women: Biased information gathering when working alongside gender and sexual minorities

**DOI:** 10.1002/jclp.23140

**Published:** 2021-05-04

**Authors:** Annalisa Anzani, Simona Sacchi, Antonio Prunas

**Affiliations:** ^1^ Department of Psychology University of Milano ‐ Bicocca Milan Italy

**Keywords:** cisgenderism, heterosexism, LGBT health, microaggressions, minority stress

## Abstract

**Objective:**

Microaggressions, a concept originally introduced for ethnic minorities, represent subtle day‐to‐day discrimination, damaging the psychological health and well‐being of lesbian, gay, bisexual, and transgender individuals as well. This study aimed to assess whether microaggressions occur in psychotherapists’ assessments of clients who identify as either lesbian or transgender woman when compared with those identifying as heterosexual woman.

**Methods:**

The study included a sample of 135 licensed psychotherapists (110 cisgender women and 25 cisgender men). Participants were presented with an audio file of a woman introducing herself during her first therapy session. Three versions were presented: a transgender, a lesbian, and a heterosexual client. Participants were asked to assess the clinical relevance of 10 questions defined as neutral (*N* = 5) and microaggressive (*N* = 5), used to determine a clinical impression of the client. A repeated measure analysis of variance was conducted to understand the likelihood of clients of different gender identity and sexual orientation receiving microaggressions.

**Results:**

Participants were more prone to consider microaggressive questions relevant where the client identified as either lesbian or transgender.

**Conclusions:**

The findings highlighted a bias against lesbian and trans women in evaluating the relevance and usefulness of clinical information while making a psychological assessment of a case.

## 1 INTRODUCTION

Health disparities and barriers in accessing care for lesbian, gay, bisexual, and transgender (LGBT) individuals are well documented in the literature (Addis et al., 2009; Burgess et al., 2007; Cochran & Mays, 2000; Dhejne et al., 2016; McCarthy et al., 2014; Seelman et al., 2017). Prejudice and bias are sustained by the heterosexist and cisgenderist systems of beliefs, which invalidate or pathologize sexual and gender minorities (Ansara & Hegarty, 2012). The Minority Stress Model helps to explain the higher risk of adverse mental health outcomes among LGBT individuals when compared with the general population (Meyer, 2003). Minority stress theorization posits that individuals with marginalized identities (such as LGBT individuals) experience unique daily stressors based on their marginalized status. Minority stressors may be correlated to external events (distal stressors) or may occur because of self‐identification as an LGBT individual (proximal stressors) (Meyer, 2003).

Microaggression theory became part of the conceptual framework emerging from the minority stress approach to account for day‐to‐day insidious discrimination. Microaggressions are described as “brief and commonplace daily verbal, behavioral, and environmental indignities, whether intentional or unintentional, that communicate hostile, derogatory, or negative insults to the target person or group” (Torino et al., 2019). These subtle forms of prejudice have a detrimental impact on the mental health and well‐being of LGBT individuals (Owen et al., 2019). Comments such as “You're just too sensitive! I'm not transphobic” or “You talk about discrimination all the time” are examples of microinvalidations that deny the personal experience, emotions, or thoughts of an individual (Anzani, 2019).

Research on microaggressions towards gender and sexual minorities often conflated transgender and LGB individuals within the broader category of the lesbian, gay, bisexual, transgender, and queer population (Anzani, 2019). Although lesbian, gay, bisexual, and transgender people's experiences of microaggressions share some common aspects, it is inappropriate to overlap them entirely. For instance, whilst LGBT individuals can experience social exclusion and poor treatment, and may feel tokenized or exoticized (Torino et al., 2019), some microaggressions may target an individual more specifically due to their sexual identity^1^ or involve issues that are uniquely shared by their identity groups (Fassinger & Arseneau, 2007). Also, as microaggressions are often based on stereotypes that can change across different minority groups, microaggressions can specifically target gay, lesbian, or bisexual people. Negative responses can be mediated by the salience and visibility of the marginalized identity in a specific situation. For example, a same‐sex couple may encounter adverse reactions from others when walking together in public, whereas a transgender individual might “pass” for being cisgender.^2^

It is also worth noting that transgender individuals might be more exposed to systemic microaggressions and forced coming‐out. It is pertinent here to consider mental health as an example where persistent systemic microaggressions occur. Although LGBT individuals share a similar history of institutionalized discrimination, gender dysphoria remains a mental health condition in the Diagnostic and Statistical Manual of Mental Disorders (DSM‐5) (APA, 2013; Fassinger & Arseneau, 2007). In an effort to depathologize trans identities, the ICD‐11 (WHO, 2018) introduced the term “Gender Incongruence,” and removed all trans‐related categories from the chapter on mental disorders (Chapter 6) to include them in a new chapter (Chapter 17) entitled “Conditions relating to sexual health.”

Transgender people also experience situations in which they necessarily have to come out (such as medical examinations), which can expose them to an increased risk of discrimination and its consequences (FRA – European Union for Fundamental Rights, 2014; Mizock & Lundquist, 2016). Research shows that among LGBT minorities, transgender individuals report the highest levels of discrimination in different social contexts, including accessing health services (FRA – European Union for Fundamental Rights, 2014). Nadal et al. (2012) defined unique aspects of transgender‐specific microaggressions. The authors identified a taxonomy of 12 transgender experience‐related microaggressions: (1) using transphobic or incorrectly gendered terminology; (2) the assumption of a universal transgender experience; (3) exoticization; (4) discomfort or disapproval of the transgender experience; (5) endorsement of gender‐normative and binary culture or behavior; (6) denial of the existence of transphobia; (7) an assumption of pathology or abnormality; (8) physical threat; (9) denial of individual transphobia; (10) denial of bodily privacy; (11) familiar microaggressions; and (12) systemic microaggressions (Nadal et al., 2012).

Despite increased mental health disparities among LGBT individuals, psychological services are mostly ineffective in meeting the needs of this specific population. Furthermore, therapeutic relationships are not immune to societal norms and values that pervade attitudes toward sex and gender (Anzani et al., 2020). Thus, although microaggressions that occur in therapy have been recognized as a violation of ethical guidelines, counseling interventions with people of marginalized groups often entail individuals being involved in a therapeutic relationship that places members in either the dominant or the marginalized social group (Morris et al., 2019). For example, if the therapist has a normative cisgender heterosexual identity, the client (by default) has a minority sexual orientation or gender identity (Sue & Sue, 2012). Microaggressions perpetrated in a therapeutic context have been found to have a negative impact in terms of outcomes, with a higher risk of drop‐out from treatment and compromised therapeutic alliance (Owen et al., 2011; Sue & Sue, 2012). Negative experiences in healthcare settings have been linked to decreased treatment satisfaction and reduction of future help‐seeking behaviors among transgender individuals (Seelman et al., 2017). Among lesbian, gay, and bisexual individuals, microaggressions in therapeutic settings have also been associated with negative emotional reactions and a change in attitudes towards psychotherapy in general (Shelton & Delgado‐Romero, 2011). Thus, further investigation of gender identity‐based microaggressions in therapeutic contexts is warranted.

Although a burgeoning area of research in recent years has been focused on microaggression towards LGBT individuals and barriers that prevent them from accessing health care, studies on microaggressions have mostly focused on the reported experiences of LGBT individuals, and no studies have investigated “in vivo” microaggressions directed toward sexual and gender minorities using a sample of experienced clinicians (Morris et al., 2019; Shelton & Delgado‐Romero, 2011). Furthermore, to the best of our knowledge, no study to date has measured psychotherapist microaggressions “in vivo” within a fictitious clinical scenario.

Another relevant issue is related to the role of emotions during clinical practice with sexual and gender minorities. Research on the emotional aspects of treatment showed that affective states could be a valid tool in making psychotherapy more effective (Plutchik, 2000). Indeed, the clinicians’ identification and expression of emotions during psychotherapy might increase clinical outcomes (Gard & Gyllensten, 2000; Gard & Lundvik Gyllensten, 2004). On the contrary, avoidance and limited understanding of clinician's own emotions are likely to have a negative impact on the therapeutic relationship and treatment. These emotional components of the therapeutic interaction could be particularly challenging during the clinical practice with LGBT individuals. As underlined by Potter et al. (2008), clinicians may experience a wide variety of emotions (i.e., anxiety, guilt, confusion, anger, or disgust) in interaction with LGBT clients, depending on their personal comfort with gender and sexual diversity, awareness, and specific skills. Furthermore, beyond the direct role played by positive and negative emotions, as well as their acknowledgment, a robust strand of research revealed a critical interaction between affective states and a broad range of socio‐cognitive processes, including attention, memory, impression formation, and behavioral responses (Isbell & Lair, 2013). More specifically, as shown by prior studies, regardless of the valence of the emotion, emotional arousal might interfere with the individual's processing capacity, thus encouraging reliance on dominant and automatic response strategies. For instance, positive affects foster simplifying heuristics and reduce motivation to elaborate information carefully. On the other side, since negative emotions, as anger, fear, and anxiety, are cognitively demanding, they limit the individual's attentional and cognitive resources, thus adversely affecting individuals’ ability to process information. Thus, differently valenced affective experiences might generate similar detrimental effects (Bodenhausen, 1993; Bodenhausen, Kramer, et al., 1994; Bodenhausen, Sheppard, et al., 1994; Isbell & Lair, 2013). Importantly, for the aim of the present work, such interference of emotions with motivation and cognitive ability might lead people to rely on stereotypic preconceptions in making social judgments (Bodenhausen, 1993). In fact, stereotypes and category‐based knowledge can be considered as a form of judgmental heuristics used by the social perceiver to simplify the social perception process and organize the behavioral response towards other people.

For these reasons, emotional arousal is generally associated with the use of simplified social schema and stereotypes that are not actively controlled or inhibited by the individual (Bodenhausen, 1993; Kim & Baron, 1988; Macrae et al., 1994). Such a process could be particularly harmful in a clinical setting involving LGBT patients given the critical role of emotions in psychotherapy and stereotypes activated in the clinicians during clinical encounter with a member of a marginalized social group.

Based on these premises, the present study aims to explore variables that predict microaggressions when psychotherapists are presented with a client whose gender or sexual identity belongs to a marginalized group, compared with a normative identity. We expect clinicians to give more relevance to microaggressive questions for a patient belonging to a sexual or gender minority group than a cisgender straight client presenting with the same clinical concerns and symptoms. Here, microaggressions will specifically refer to the (undue) link of the patient's gender identity or sexual orientation to the problematic or symptomatic aspects presented. We also expect that the self‐assessed emotional arousal generated by a patient belonging to a marginalized group will moderate the relevance attributed to microaggressive questions for the transgender and lesbian condition. Moreover, starting from the evidence of the relation between emotional arousal and automatic responses, we hypothesized highly activated clinicians to be more prone to use microaggressions toward sexual and gender minorities than clinicians at moderate levels of arousal.

## 2 PILOT STUDY

To test whether psychotherapists could enact microaggressions in the context of the first consultation with a client, we had to create ad hoc questions that were relevant to the presented case. For this purpose, we asked 84 early career psychologists to listen to the audio files, that were then used in the main study, and produce up to 10 questions they would have asked the client to have a better understanding of the clinical case.

Participants generated 575 questions in total, with an average of 6.8 questions each. Three different raters (blind to the study's hypotheses) independently classified each of the items as nonmicroaggressive, possibly microaggressive, or microaggressive. The raters were trained to recognize microaggressions; they were provided with the definition of microaggressions several examples of microaggressions targeting sexual and gender minorities. Approximately 30.9% of the sample produced at least one microaggressive question (rated as microaggression by two out of three raters). Only the questions from the original pool of items on which all three raters agreed upon were used to construct the questions for the following study.

## 3 CURRENT STUDY

### 3.1 Methods

#### 3.1.1 Participants

An a priori power analysis was conducted to estimate the required sample size using G*Power (Faul et al., 2007). The power analysis (alpha = 0.05, power = 0.80, effect size *η* ^2^_partial_ = 0.25) suggested a sample size of *N* = 120 for a 3 between‐participants × 2 within‐participants analysis of variance (ANOVA). In total, 135 licensed psychotherapists took part in the study, all of whom self‐identified as cisgender, White Italian citizens, with 110 (81.5%) identifying as female. Other demographic characteristics of the sample are summarized in Table 1.

**Table 1:** Demographic characteristics of the sample

Characteristics	Total	Female	Male
	*N* = 135	*N* = 110	*N* = 25
	*N* (%)	*N* (%)	*N* (%)
Age groups (years)			
27–35	32 (23.7)	28 (25.5)	4 (16)
36–45	54 (40)	41 (37.3)	13 (52)
46–55	30 (22.2)	27 (24.5)	3 12)
56–65	14 (10.4)	10 (9.1)	4 (16)
65+	5 (3.7)	4 (3.6)	1 (4)
Relationship status			
Single	45 (33.3)	37 (33.6)	8 (32)
Married	51 (37.8)	42 (38.2)	9 (36)
Divorced	5 (3.7)	4 (3.6)	1 (4)
Widow	2 (1.5)	1 (1.8)	0
Cohabitant	32 (23.7)	25 (22.7)	7 (28)
Sexual orientation			
Heterosexual	120 (88.9)	101 (91.8)	19 (76)
Gay/lesbian	4 (3)	0	4 (16)
Bisexual	10 (7.4)	8 (7.3)	2 (8)
Other	1 (0.7)	1 (0.9)	0
Degree			
Psychology	132 (97.8)	107 (97.3)	25 (100)
Medicine	1 (0.7)	1 (0.9)	0
Philosophy	2 (1.5)	2 (1.8)	0
Literature	4 (3)	4 (3.6)	0
Post‐grad education			
Master	61 (45.2)	54 (49.1)	7 (28)
Advanced training courses	37 (27.4)	29 (26.4)	8 (32)
Doctorate	8 (5.9)	4 (3.6)	4 (16)
Psychotherapy training	122 (90.4)	98 (89.1)	24 (96)
LGBT+ training	27	20 (18.2)	7 (28)
Sexology training	17 (12.6)	15 (13.6)	2 (8)
Theoretical orientation			
Cognitive‐behavioral	32 (23.7)	28 (25.5)	4 (16)
Systemic	18 (13.3)	16 (14.5)	2 (8)
Psychodynamic/psychoanalytic	55 (40.7)	43 (39.1)	12 (48)
Other	30 (22.2)	23 (20.9)	7 (28)
Years of practice			
1–5	49 (36.3)	42 (38.2)	7 (28)
6–10	33 (24.4)	24 (21.8)	9 (36)
11–20	31 (23)	26 (23.6)	5 (20)
21–30	18 (13.3)	15 (13.6)	3 (12)
30+	4 (3)	3 (2.7)	1 (4)

#### 3.1.2 Procedure

The experiment was conducted following the guidelines defined by the Declaration of Helsinki. The study was approved by the local ethics committee, and informed consent was obtained from all participants. The regional boards of psychologists and psychiatrists across the country were contacted to achieve the highest possible visibility of this study project among their members. Therapists were contacted by email with brief details about the online study and an invitation to participate voluntarily and were provided with a direct link to the online survey administered via Qualtrics. Participants were asked to participate in a study aimed at understanding the behavior of psychotherapists when facing complex cases, in terms of diagnostic framework and prognosis. They were first presented with an audio file of a woman who identified as either transgender, cisgender lesbian, or cisgender heterosexual (depending on the condition) introducing herself and describing her problems (related to panic) during the first session with the participant (see Appendix 1). The audio recording was performed by a professional actress who played all three roles. To make the scenes as similar as possible, we edited the audio file so that only the first recorded phrase differed for the three conditions. The procedure was conducted entirely online, and participants were randomly assigned to one of the three experimental conditions. After listening carefully to the audio file, therapists were asked to complete a questionnaire. Detailed information about their training and clinical experience was also collected (see Table 1). The survey was completed anonymously, and, at the end of the procedure, participants were thanked and debriefed.

#### 3.1.3 Measures

##### 3.1.3.1 Microaggressive and neutral questions

We first asked therapists to evaluate the relevance of 10 questions they might have asked the client to obtain a full clinical assessment, on a scale from 1 (*not relevant at all*) to 7 (*extremely relevant*). The questions reflected both microaggressive and neutral themes. The questions generated from the pilot study were used to build five microaggressive (Cronbach's *α* = 0.90) and five neutral questions (Cronbach's *α* = 0.71) for the current study (questions are presented in Appendix 2). In general, microaggressive items were related to the client's sexual identity (e.g., “How was discovering your sexual identity for you?”). In contrast, neutral questions were about clinical symptoms and complaints, clinical history, and other clinically relevant aspects mentioned by the client in the presentation (e.g., “How frequent are the panic attacks you described?”).

##### 3.1.3.2 The Positive and Negative Affect Schedule

This is a 20‐item mood scale that comprises both positive and negative effects. The scale was administered immediately after the listening task. We used the scale to compute a global score of emotional arousals. The Cronbach's alpha for the global scale was *α* = 0.78 (Terracciano et al., 2003; Watson et al., 1988).

#### 3.1.4 Statistical analyses

To compare the relevance of microaggressive and neutral questions depending on the sexual identity of the client, we computed a 3 × 2 ANOVA model. To ascertain whether emotional arousal influenced the relevance attributed to microaggressive questions, a moderation model was carried out through PROCESS (model 1, 5000 bootstrap resampling) (Hayes, 2018). Finally, to ascertain whether the therapists’ individual differences influenced their responses, we controlled for the effect of the therapists’ gender and sexual orientation through ANOVAs.

## 4 RESULTS

### 4.1 The relevance of microaggressive versus neutral questions

To compare the relevance of microaggressive and neutral questions depending on the sexual identity of the client, we computed a 3 (condition: heterosexual, lesbian, and transgender) × 2 (type of questions: microaggressive and neutral) ANOVA with the first‐factor as between‐participants and the second‐factor as within‐participants.

**Figure 1:**
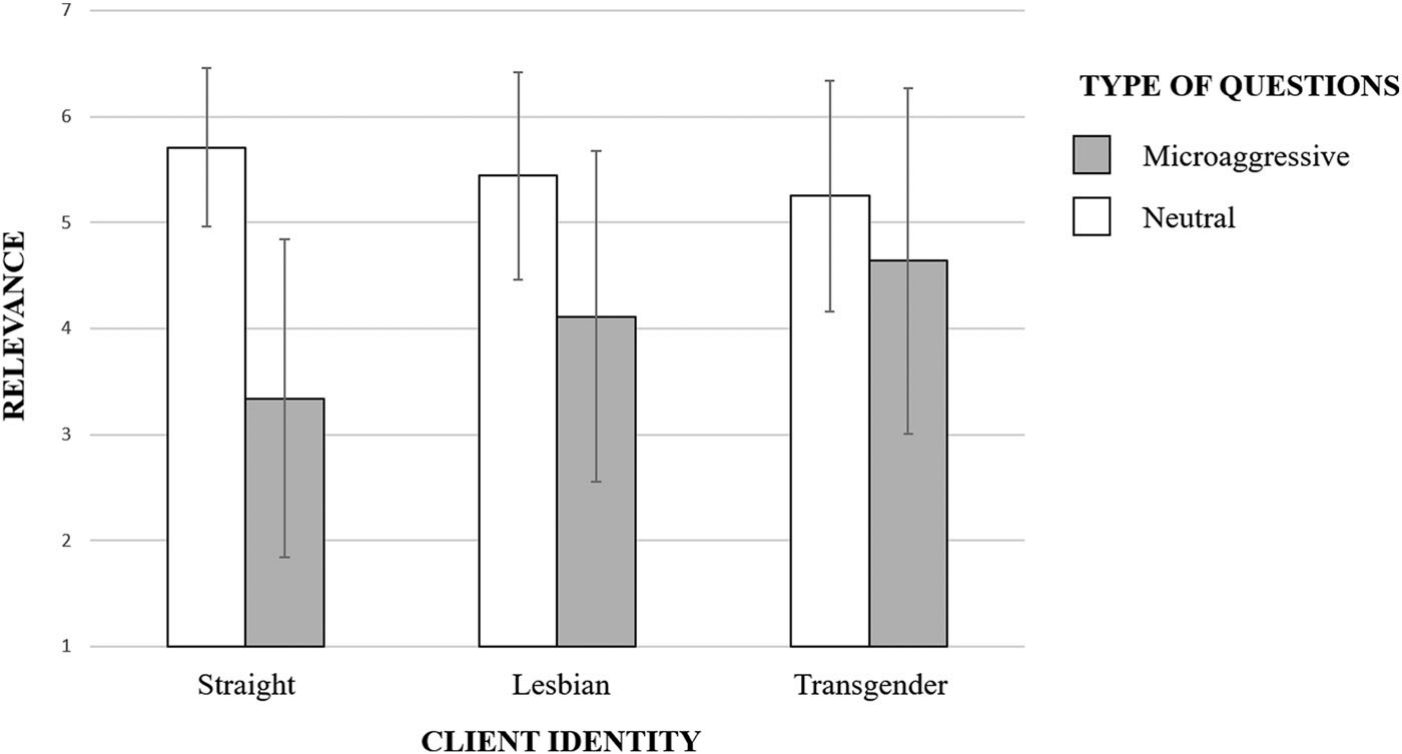
The interaction effect between condition and type of questions. As displayed, neutral questions were rated more relevant in the cisgender straight client condition, than in the transgender client condition. The analysis revealed no differences between cisgender lesbian and transgender conditions, and between cisgender straight and lesbian conditions

The analysis suggested a main effect for the type of questions: *F* (1, 132) = 119.36, *p* < 0.001, *η*_p_^2^ = 0.47. Independent from the experimental condition, neutral questions were considered significantly more relevant (*M* = 5.44, *SD* = 0.98) than microaggressive questions (*M* = 4.09, *SD* = 1.63). The ANOVA did not yield a significant main effect of condition: *F* (1, 132) = 1.76, *p* = 0.18, *η*_p_^2^ = 0.03. However, in line with the hypothesis, a significant interaction effect between condition and type of questions emerged: *F* (2, 132) = 14.56, *p* < 0.001, *η*_p_^2^ = 0.18. As displayed in Figure 1, neutral questions were rated more relevant in the cisgender straight client condition (*M* = 5.70, *SD* = 0.75), than in the transgender client condition (*M* = 5.25, *SD* = 1.09), *t*(86) = 2.22, *p* = 0.03, *d* = 0.48, 95% confidence interval [CI] [0.05–0.90]. The analysis revealed no differences between cisgender lesbian (*M* = 5.44, *SD* = 0.98) and transgender conditions, *t*(95) = 0.92, *p* = 0.36, and between cisgender straight and lesbian conditions, *t*(83) = 1.36, *p* = 0.18. The post hoc comparisons of the relevance of microaggressive questions showed no difference between the cisgender lesbian (*M* = 4.11, *SD* = 1.59) and transgender conditions (*M* = 4.64, *SD* = 1.56), *t*(95) = 1.68, *p* = 0.09. Crucially, microaggressive questions were rated as significantly less relevant for the cisgender straight (*M* = 3.34, *SD* = 1.50) than for the cisgender lesbian client, *t*(83) = 2.26, *p* = 0.03, *d* = 0.49, 95% CI [0.06–0.93], and the transgender client, *t*(86) = 3.94, *p* < 0.001, *d* = 0.85, 95% CI [0.41–1.29].

### 4.2 The effect of the emotional arousal

**Figure 2:**
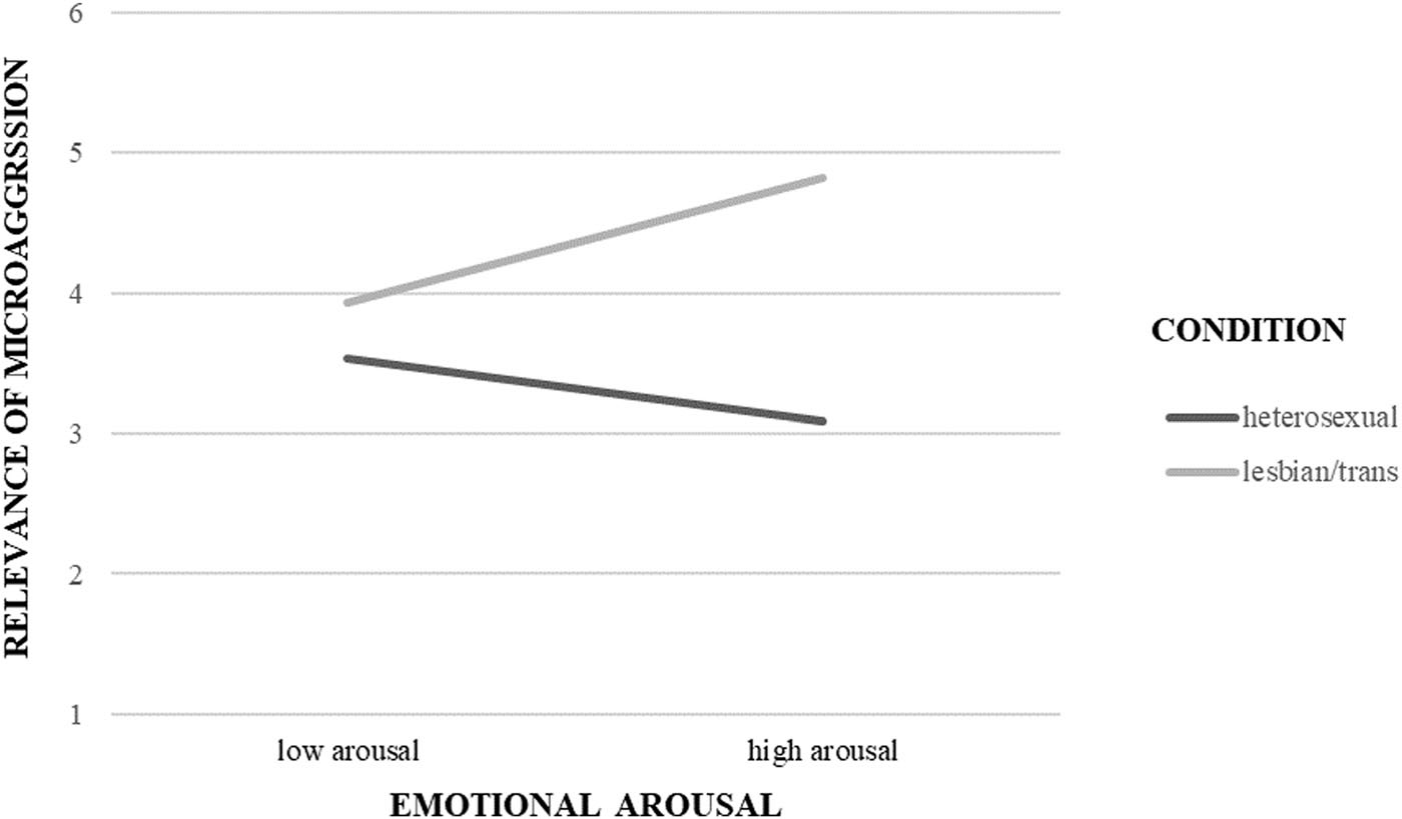
The interaction effect between condition (cisgender straight woman = 0, cisgender lesbian and transgender woman = 1) and emotional arousal on the relevance of microaggressive questions

To ascertain whether emotional arousal influenced the relevance attributed to microaggressive questions, a moderation model was carried out through PROCESS (model 1, 5000 bootstrap resampling) (Hayes, 2018). In this model, we used the experimental condition (cisgender straight woman = 0, cisgender lesbian and transgender woman = 1) as the independent variable (X), the therapist's emotional arousal as moderator (W), and the relevance of microaggressive questions as the dependent variable (Y). We chose emotional arousal rather than the valence of emotions for two reasons. A methodological reason relies on the fact that the Positive and Negative Affect Schedule (PANAS) demonstrated good internal consistency (*α* = 0.78), suggesting that both positive and negative items of the scale measured the same construct. A more theoretical reason is based on the argument that the positive affect and negative affect subscales of the PANAS both measure the latent construct of emotional arousal (Russell et al., 1989). Because the relevance of microaggressive questions did not differ between the two conditions, we decided to collapse the conditions for both the cisgender lesbian and the transgender clients. As showed in Figure 2, the interaction effect between condition and arousal on the relevance of microaggressive questions was significant (*B* = 1.90, *SE* = 0.82, *t* = 2.32, *p* = 0.02, 95% CI [0.28–3.53]). At a high level of arousal ( = 2.55), therapists rated microaggressive questions more relevant for cisgender lesbian and transgender women than for cisgender straight women (*B* = 1.74, *SE* = 0.43, *t* = 4.04, *p* < 0.001, 95% CI [0.89–2.59]). At low levels of arousal ( = 1.85), the difference between cisgender straight and cisgender lesbian/transgender conditions was not significant (*B* = 0.40, *SE* = 39, *t* = 1.04, *p* = 0.30, CI [−0.37 to 1.17]).

### 4.3 Individual differences

To ascertain whether the therapists’ individual differences influenced their responses, we controlled for the effect of the therapists’ gender and sexual orientation. The first ANOVA yielded no significant main effect for gender, *F* (1, 129) = 0.001, *p* = 0.97, or any interaction effects with gender (*Fs* < 0.63, *ps* > 0.43). The same results were obtained introducing sexual orientation as an independent variable (*Fs* < 0.95, *ps* > 0.33). We also controlled for the effect of the therapists’ theoretical background (cognitive‐behavioral, systemic, psychodynamic, or other) and years of practice on relevance evaluations. No interaction or main effects of theoretical background were identified (*Fs* < 1.25, *ps* > 0.29), or for years of practice (*Fs* < 0.87, *ps* > 0.54).

## 5 DISCUSSION

The aim of this study was to investigate the presence of microaggressive responses when psychotherapists work with clients of a marginalized sexual orientation or gender identity (compared with a normative sexual orientation and gender identity). The findings highlighted a bias in evaluating the relevance and usefulness of information while making a psychological assessment of a case. Data showed the effect of the client's gender identity and sexual orientation in rating the relevance of microaggressive questions by the therapists. Such questions were considered less important for the normative straight cisgender client and more important for the less conforming clients (cisgender lesbian and transgender). Conversely, asking general questions about symptoms, life events, and mental health history was considered less relevant for the assessment in the transgender client condition compared to the cisgender straight client condition.

Results indicated that both cisgender lesbian and transgender women were equally targets of microaggressions. In addition, neutral questions were considered significantly less relevant only in the transgender condition compared with the cisgender straight condition. This result could be explained by considering the long history of pathologization of transgender identities. Once a client is labeled as transgender, they are immediately “diagnosed” by some of the psychotherapists that probably still rely on a pathologizing understanding of transgender identities. Thus, the main issues and complaints they bring to the consultation fade into the background as the therapist no longer needs additional information. Such bias relies on specific assumptions based on cisgenderism and heteronormativity (Ansara & Hegarty, 2012; Lev, 2013).

First therapists may ask more questions on the sexual identity of cisgender lesbian and transgender clients because they connect the patients’ symptoms with the patients’ sexual orientation and gender identity (Anzani et al., 2020). The assumption that sexual identity is the cause of their current symptoms contributes to the idea being perpetuated that sexual and gender marginalized identities are inherently “wrong” and problematic, fostering internalization of negative attitudes towards one's own minority identity. The second fundamental assumption that influences therapists to ask questions about sexual identity relies on stereotypical beliefs about LGBT experiences. Questions regarding how sexual identity was “discovered” and how LGBT individuals manage their sexuality in different social and relational contexts are based on stereotypical assumptions. For example, assuming that sexual identity is a discovery (or a choice) that causes difficulties for the individual is a narrative based on a stereotypical representation of LGBT individuals that does not necessarily reflect the experience of every member of the community. Thus, cisgenderist and heterosexist paradigms still appear to have a significant impact in shaping people's attitudes towards sexual and gender minorities, including among mental health professionals.

Those therapists more emotionally aroused were more prone to be microaggressive with a marginalized identity than therapists that showed low levels of arousal. The literature suggests that there is a relationship between emotional arousal and/or anxiety and prejudice and stereotypes (Britt et al., 1996; Vescio & Biernat, 1999). Emotional arousal interferes with the psychotherapists’ process of cognitive evaluation and impression formation. As a result, psychotherapists have fewer cognitive resources at their disposal and to rely more on biases and stereotypes in making judgments (Bodenhausen, 1993). Thus, when confronted with a member of a marginalized group, some psychotherapists might activate stereotypes that lead to emotional arousal directly detrimental to their judgment. For example, arousal has been shown to reduce the availability of relevant information caused by a narrowed attentional focus (Easterbrook, 1959).

The main guidelines for psychological practice with LGBT clients promote understanding, respect, and support, recognizing the existence of minority stress as an essential factor affecting the mental health of LGBT individuals (American Psychological Association, 2012, 2015; Coleman et al., 2012; Morris et al., 2019). Despite the guidelines that ensure care and protection for the LGBT population, some therapists continue to demonstrate bias when confronted with a member of the community as a client (Prunas et al., 2018). As psychologists and psychotherapists, our purpose is to take care of our client's mental health and well‐being. From this perspective, trying to reduce this bias is crucial to be able to fight health barriers and disparities.

A strong proactive strategy would be to endorse a paradigm shift from microaggressions to microaffirmations, clearly helpful in working towards providing real support and affirmation of LGBT identities (Anzani, Morris, et al., 2019). The first step in this direction could be made by providing adequate knowledge and developing competence among therapists on LGBT issues. The Italian educational system provides no adequate training on gender, sexual diversity, and sexual or gender minorities’ needs for healthcare professionals. As a result, it is likely that psychologists and healthcare providers in general are still affected by common thinking and societal paradigms rather than focusing on knowledge and skills. A strongly affirmative and supportive practice must be based on adequate and specific training. The microaffirmation model of care recognizes affirmative practices such as the acknowledgment of cisnormativity and its disruption as beneficial. Such practice starts primarily by acknowledging differences in privilege based on the identities of people involved in the therapeutic relationship through an intersectional perspective.

### 5.1 Limitations

The study suffers from several limitations. First, to make results comparable across minority groups, we focused exclusively on a specific type of microaggression particularly relevant in clinical practice, which consists of establishing a causal connection between the symptoms and problems presented by the client with their gender and sexual identity. Future research could further explore more broadly the specific differences that may exist in the whole array of microaggressions against LBG and transgender people in clinical practice. Moreover, we did not make explicit the sexual orientation of the transgender client during her presentation. Therefore, the comparison of the three experimental conditions is slightly unbalanced given that this piece of information was not provided. In addition, future studies may consider microaggressions in clinical practice against other sexual and gender minorities (i.e., bisexual or nonbinary people).

## 6 CONCLUSIONS

In spite of these limitations, the study presents numerous strengths, including investigating microaggressions directed toward gender/sexual minorities “in vivo,” with a sample of licensed and experienced psychotherapists. This approach, rather than relying on the reported experiences of LGBT patients in therapy, allows observing directly the therapists' response to the client's disclosure of information. The use of an audio file in which the client introduces herself identifying as lesbian, trans, or heterosexual, allows the understanding of the intrinsic and implicit functioning of microaggressions through the approach of the therapist. Furthermore, the inclusion of emotional arousal among the study variables allows spreading lights on other dimensions that can potentially be targeted in the training of mental health professionals on LGBT issues.

### PEER REVIEW

The peer review history for this article is available at https://publons.com/publon/10.1002/jclp.23140

## Data Availability

The data presented in this study are available on request from the corresponding author. The data are not publicly available due to privacy reasons.

## ENDNOTES

1 Sexual identity is a complex construct that reflects how an individual perceives themselves on four dimensions: sex assigned at birth, gender identity, sexual orientation and, gender role (Shively & De Cecco, 1977; Sell, 1997). Throughout the paper, we will refer to sexual identity as a broad construct that includes and integrates those four different dimensions.

2 “Passing” is an expression used to indicate when someone, typically a transgender person, is perceived as a cisgender person of the gender they identify with, instead of a person of the gender they were assigned at birth. This might be the case, for example, of a trans woman who is perceived as a cisgender woman. A trans person who is perceived as cisgender may face less prejudice, harassment, and risk of violence. However, the appropriateness of the term “passing” is debated within the transgender community.

## APPENDIX 1 TRANSCRIPT OF THE AUDIOFILE PRESENTED TO THE PSYCHOTHERAPISTS (ENGLISH VERSION)^3^

Hi, my name is Elisa, I am a 34‐year‐old straight/lesbian/trans woman. I got a degree in industrial design and now I work as a fashion journalist. I have been single for about 2 years now. I live with my mother, who suffers from Alzheimer's disease. I am here because of panic attacks which first appeared around a year ago, and they have increased in frequency ever since. Currently, they might occur up to two or three times a day. The attacks start with a sudden wave of fear, coming out of the blue, most often during the day but sometimes during sleep or when I wake up. I start trembling, I feel nausea, and sweat all over my body, I can't breathe and I am afraid I am losing control of my actions. The first attack appeared last year, when the head of the company I work for changed, and for a few weeks I feared I would lose my job, being replaced by a less‐qualified colleague supported by the new director. Sometimes up to 2 weeks can pass between one attack and the next, it often happens when I am more relaxed, but life stressors have a significant impact.

## APPENDIX 2 MICROAGGRESSIVE AND NEUTRAL QUESTIONS PRESENTED TO THE PSYCHOTERAPISTS^3^

Microaggressive questions

- How was discovering your sexual identity for you?
- How do you experience your sexual identity in relation to your coworkers/friends?
- To what extent your sexual identity could affect panic attacks?
- What is your mother's position about your sexual identity?
- Have you ever had any problems with your sexual identity?

Neutral questions

- How many years has your mother been sick with Alzheimer's disease?
- Is this the first time you have asked a mental health professional for help?
- What is your career path?
- Did you receive any help from your relatives or friends in taking care of your mother?
- How frequent are the panic attacks you described?
